# Njimo Wood

**Published:** 1887-08

**Authors:** 


Njimo Wood.—Njimo Wood is a new drug from Western Africa, derived from Sarcocephalus Esculentus, one of the Cinchonaceæ, which may possibly find a place in the materia medica. The natives use it in all cases of gastric troubles, loss of appetite, etc., and it furnishes, similarly to the Coca leaves of America, a pleasant stimulant much in request with the negroes.

Experiments made with an extract obtained from the wool, with a view to determining the amount of alkaloid, did not succeed; a resin and bitter principle only were isolated.—*Pacific Record*, April 15, 1887.

